# HAT_2_CH_2_ score performance predicting neurologic events after cardiac implantable electronic device

**DOI:** 10.7150/ijms.72497

**Published:** 2022-05-21

**Authors:** Ju-Yi Chen, Tse-Wei Chen, Wei-Da Lu

**Affiliations:** Department of Internal Medicine, National Cheng Kung University Hospital, College of Medicine, National Cheng Kung University, Tainan, Taiwan

**Keywords:** Atrial high-rate episodes, cardiac implantable electronic device, HAT_2_CH_2_ score, neurologic events

## Abstract

**Objectives:** The HAT_2_CH_2_ score has been evaluated for predicting new-onset atrial fibrillation in several clinical conditions, but never for adverse neurologic events. We aimed to evaluate the effectiveness of HAT_2_CH_2_ score in predicting neurologic events in patients with cardiac implantable electronic device (CIED), comparing with atrial high-rate episodes (AHRE).

**Methods:** This case-control study enrolled 314 consecutive patients aged 18 years or older with CIED implantation between January 2015 and April 2021. Patient data were analyzed retrospectively. The primary endpoint was subsequent neurologic events (NE) after implantation. AHRE was defined as > 175 bpm (Medtronic®) lasting ≥ 30 seconds. Variables associated with independent risk of NE were identified using multivariate Cox regression analysis with time-dependent covariates.

**Results:** Patients' median age was 73 years and 61.8% of them were male. During follow-up (median 32 months), 18 NE occurred (incidence rate 2.15/100 patient-years, 95% CI 1.32-4.30). Multiple Cox regression analysis showed that the HAT_2_CH_2_ score (HR 2.424, 95% CI 1.683 - 3.492, p < 0.001) was an independent predictor for NE. Optimal HAT_2_CH_2_ score cutoff value was 3 with highest Youden index (AUC, 0.923; 95% CI, 0.881-0.966; p < 0.001). Both AHRE ≥ 1 minute and HAT_2_CH_2_ score ≥ 3 had the highest AUC of the receiver-operating characteristic (0.898, 95% CI, 0.831-0.965, p < 0.001). Significant increase was observed in NE occurrence rates using the HAT_2_CH_2_ score (p < 0.001).

**Conclusion:** The HAT_2_CH_2_ score and episodes of AHRE lasting ≥ 1 minute are independent risk factors for NE in patients with CIED.

## Introduction

The HAT_2_CH_2_ score, which comprises hypertension <1 point>, age >75 years <1 point>, stroke or transient ischemic attack <2 points>, chronic obstructive pulmonary disease [COPD] <1 point>, and heart failure <2 points>, was first developed in 2010 to identify patients likely to progress to sustained forms of atrial fibrillation (AF) in the near future 1. Since then, numerous studies have been conducted to examine the prediction of AF in several clinical conditions, including post ablation for atrial flutter 2, cancer patients 3, after coronary bypass surgery 4, patients receiving electric cardioversion 5, and emergency-department patients 6. Recently, the C_2_HEST score 7 and mCHEST score 8 were also evaluated for predicting new AF, which revealed acceptable discriminating power. However, the predictive performance of the HAT_2_CH_2_ score, C_2_HEST score and mCHEST score for new-onset atrial fibrillation and neurologic events (NE) in patients with cardiac implantable electronic device (CIED) was rarely evaluated.

The latest European Society of Cardiology (ESC) guidelines regarding non-valvular AF 9 state that AHRE > 5-6 minutes and > 180 bpm detected by CIED increase the risk for NE and clearly recommend that AHRE should be closely monitored and treated. The CHA_2_DS_2_-VASc score is also recommended for risk stratification of NE 10. However, the utility of CHA_2_DS_2_-VASc in non-valvular AF patients is debated and controversial, primarily because it is a vascular scoring system, which does not incorporate AF-related parameters 10. Meta-analysis showed that the discriminative power of the CHA_2_DS_2_-VASc score was only modest in predicting NE, and results were similar in the presence or absence of non-valvular AF 10. HASBLED score is recommended to use as a bleeding risk score 9, however, never to be used to predict NE. Also, the performance of several scoring systems (CHA_2_DS_2_-VASc score, HAT_2_CH_2_ score, C_2_HEST score and mCHEST score) and the optimal cutoff for AHRE duration to predict subsequent NE in patients with CIED have not been well studied previously. Therefore, more accurate NE prediction models are still needed for the non-valvular AF population and patients with CIED.

The present study aimed to investigate the performance of the HAT_2_CH_2_ score in predicting NE compared to AHRE in minutes and other scoring systems (CHA_2_DS_2_-VASc score, HASBLED score, C_2_HEST score and mCHEST score) in patients with CIED and without a history of AF.

## Methods

Consecutive patients aged 18 years or older who underwent CIED implantation (Medtronic®: dual chamber pacemaker, dual chamber implantable cardioverter defibrillator, cardiac resynchronization therapy-pacing, and cardiac resynchronization therapy-defibrillator) in the Cardiology Department of National Cheng Kung University Hospital from January 2015 to April 2021 were included. All patient data were retrieved from hospital records and were analyzed retrospectively.

### Ethical considerations

The protocol for this cohort study was reviewed and approved by the ethics committee of National Cheng Kung University Hospital and was conducted according to guidelines of the International Conference on Harmonization for Good Clinical Practice (B-ER-108-278). All included patients provided signed informed consent at the time of their implantation procedures for data to be recorded for later publication in accordance with the ethical standards laid down in the 1964 Declaration of Helsinki and its later amendments.

### Patient and Public Involvement statement

Patients and the public did not involve in the design, or conduct, or reporting, or dissemination plans of the research.

### Data collection and definitions

Patients' medical history and data of co-morbidities and echocardiographic parameters were collected from chart records for retrospective evaluation during the implantation date and as the baseline data. Diabetes mellitus was defined by the presence of symptoms and casual plasma glucose concentration ≥ 200 mg/dL, fasting plasma glucose concentration ≥ 126 mg/dL, 2-hour plasma glucose concentration ≥ 200 mg/dL from a 75-g oral glucose tolerance test, or taking antidiabetic medication. Hypertension was defined as in-office systolic blood pressure values ≥ 140 mmHg and/or diastolic blood pressure values ≥ 90 mmHg or taking antihypertensive medication. Dyslipidemia was defined as low-density lipoprotein ≥ 140 mg/dL, high-density lipoprotein < 40 mg/dL, triglycerides ≥ 150 mg/dL, or taking medication for dyslipidemia. Chronic kidney disease was defined as an estimated glomerular filtration rate (eGFR) < 60 mL/ min / 1.73 m^2^ for at least 3 months.

AHRE were extracted from the devices via telemetry performed at each office visit (3~6 months). AHRE electrograms were reviewed by at least one experienced electrophysiologist, who carefully considered the possibility that AHRE included lead noise or artifacts, far-field R-waves, paroxysmal supraventricular tachycardia and visually confirmed AF in the detected AHRE. Atrial sensitivity was programmed to 0.3 mV with bipolar sensing of Medtronic devices. AHRE was defined as heart rate >175 bpm and at least 30 seconds of atrial tachyarrhythmia recorded by the devices on any day during the study period. If patients had multiple AHRE, the longest AHRE duration was recorded. The duration between data extraction and device implantation was similar in every subject. No consistent increase of AHRE in different extraction after device implantation was found.

The primary endpoint was the occurrence of NE after the date of CIED implantation, including stroke or TIA diagnosed by experienced neurologists. For each outcome, only the first event of that outcome in a specific subject was included. For the composite outcome, only the first event in a given patient was included.

### Statistical analysis

Categorical variables are presented as percentages and continuous variables as means and standard deviations (mean ± SD) for normally distributed values or medians and interquartile interval (IQI) for non-normally distributed values. The normal distribution for continuous variables was assessed using the Kolmogorov-Smirnov method. Pearson's chi-square test or Fisher's exact test was used to determine differences in baseline characteristics for categorical variables, and a two-sample student's t-test or Mann-Whitney U-test was used to analyze continuous variables. Survival was estimated by the Kaplan-Meier method, and differences in survival were evaluated with the log-rank test. Multivariable Cox regression analysis was used to identify variables associated with NE occurrence, reported as hazard ratios (HR) with 95% confidence intervals (CI). If the p value for any factor in univariable analysis was < 0.05, the parameter was entered into multivariable analysis. Because CHA_2_DS_2_-VASc scores, HASBLED scores, and HAT_2_CH_2_ scores overlapped many factors in univariate analysis, they were used as independent factors in multivariable Cox regression analysis. We assured that the proportional hazard equation was met in each variables using log minus log plot, which each curve did not meet during the observation periods. Indicators of CHA_2_DS_2_-VASc scores, HASBLED scores, and HAT_2_CH_2_ scores were used separately as time-dependent covariates in Cox proportional hazards regression, to compute hazard ratios (HR) and adjusted HR in multivariable models including clinical variables, such as prior stroke, diabetes, hyperlipidemia, and AHRE ≥ 1 minute. We used the linear regression analysis to address the multicollinearity. A tolerance of less than 0.20 or 0.10 and/or a VIF (variance inflation factor) of 5 or 10 and above indicates a multicollinearity problem. We assured that each variable selection had no multicollinearity problem. The receiver-operating characteristic (ROC) area under the curve (AUC) for AHRE and the HAT_2_CH_2_ score and the associated 95% confidence intervals (CI) were evaluated for associations with future NE after CIED implantation. The optimal cutoff values with the highest Youden index were chosen based on the results of ROC curve analysis and used to evaluate the associated values of AHRE in minutes and HAT_2_CH_2_ score for determining NE. For all comparisons, p < 0.05 was considered statistically significant. All data were analyzed using SPSS statistical package version 23.0 (SPSS Inc. Chicago, IL, USA).

## Results

Between January 1, 2014 and April, 2021, a total of 453 consecutive patients receiving Medtronic CIED transplantation at National Cheng Kung University Hospital were recruited initially. Patients with previous AF (n=139) were excluded. The final analysis included data of 314 patients, of whom 18 had experienced NE.

The median follow-up period was 32 months after implantation of CIEDs. Table 1 shows patients' baseline demographic and clinical characteristics based on the occurrence of NE or not. Patients' median age was 73 years and 61.8% of patients were men. Types of CIEDs included dual chamber pacemaker (220, 70.1%), dual chamber ICD (66, 21.0%), CRTP (23, 7.3%) and CRTD (5, 1.6%). The most common indication for CIED implantation was sick sinus syndrome (44.9%), followed by atrioventricular block (25.2%) and ventricular tachyarrhythmia (29.9%) (Table 1). Overall atrial pacing median percentages (25.0%) and ventricular pacing median percentages (1.9%) were noted. High percentages of hypertension (80.6%), hyperlipidemia (76.8%), and diabetes (45.2%) suggest relatively high risk of NE for the entire study cohort. Components of NE, incidence rates, and 95% CI are reported in Table 2. Overall, the total number of NE was 18 (incidence rate (IR) 2.15/100 patient-years, 95% CI 1.32-4.30) (Table 2).

### Univariable analysis and multivariable Cox regression analysis to identify independent predictors of NE

Univariable analysis revealed that prior stroke, diabetes mellitus, hyperlipidemia, AHRE in minutes, AHRE ≥ 1 minute, CHA_2_DS_2_-VASc score, HAS-BLED score, and HAT_2_CH_2_ score were significantly associated with NE occurrence (Table 1). Multivariable Cox regression analysis using model 1-3 in Table 3 (including CHA_2_DS_2_-VASc score, HASBLED score, and HAT_2_CH_2_ score as confounders) showed that only HAT_2_CH_2_ score (HR 2.424, 95% CI 1.683 - 3.492, p < 0.001) was independently associated with NE. When we used the all variables in CHA_2_DS_2_-VASc score and HAT_2_CH_2_ score as confounders, only prior stroke or TIA (HR 11.726, 95% CI 3.649 - 37.678, p < 0.001) was the independent factor of NE (data not shown). We also showed that the HAT_2_CH_2_ score was an independent predictor for new onset AF in Supplementary Table 1 and 2.

### ROC-AUC determination of AHRE and HAT_2_CH_2_ score cutoff values as predictive factors for future NE and survival analysis

**Figure 1:**
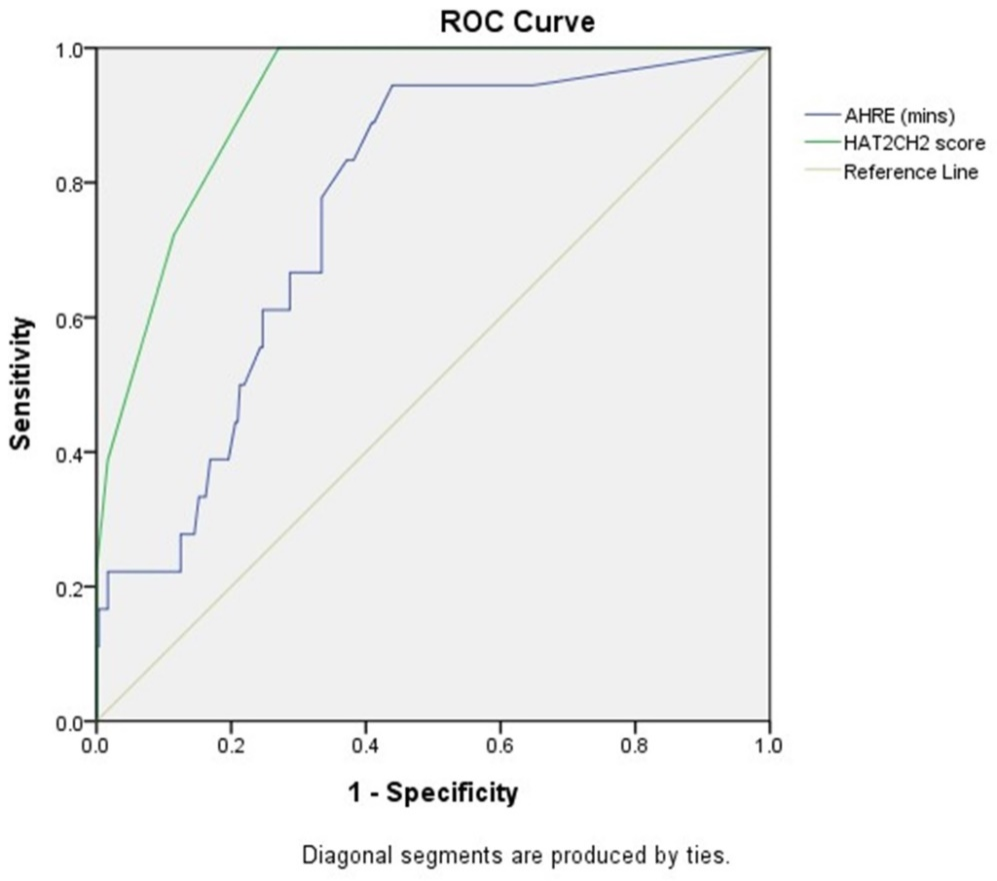
Receiver-operating characteristic curve analysis of atrial high-rate episodes (minutes) and HAT_2_CH_2_ score in patients with CIED with subsequent neurologic events. Atrial high-rate episodes (minutes): optimal cutoff value with the highest Youden index, 1 minute; sensitivity, 94.4%; specificity, 56.1%; AUC, 0.761; 95% CI, 0.664-0.857; p < 0.001. HAT_2_CH_2_ score: optimal cutoff value with the highest Youden index, 3; sensitivity, 100.0%; specificity, 73.0%; AUC, 0.923; 95% CI, 0.881-0.966; p < 0.001.

The optimal AHRE cutoff value predictive of future NE was 1 minute with the highest Youden index (sensitivity, 94.4%; specificity, 56.1%; AUC, 0.761; 95% CI, 0.664-0.857; p < 0.001) (Fig. 1). The optimal HAT_2_CH_2_ score cutoff value predictive of future NE was 3 with the highest Youden index (sensitivity, 100.0%; specificity, 73.0%; AUC, 0.923; 95% CI, 0.881-0.966; p < 0.001) (Fig. 1).

**Figure 2:**
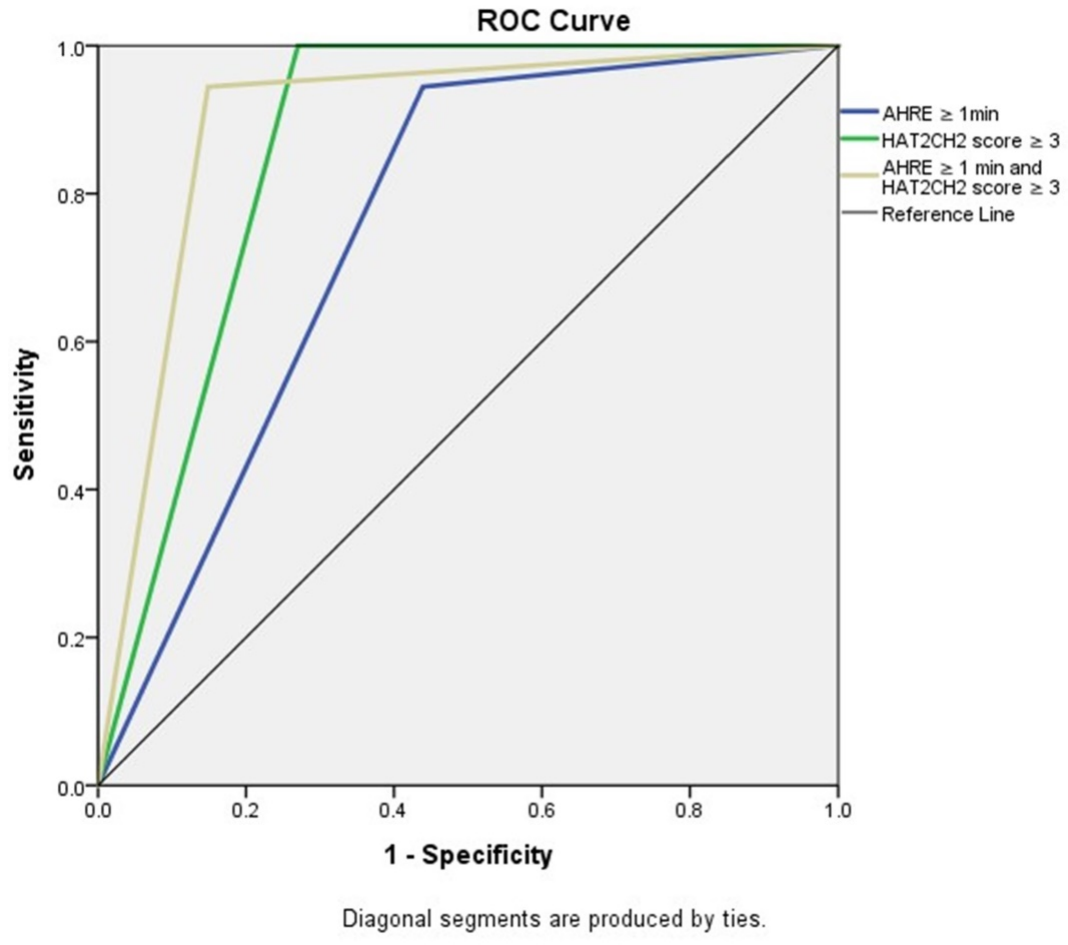
Receiver-operating characteristic curve analysis of AHRE ≥ 1 minute, HAT_2_CH_2_ score ≥ 3, and AHRE ≥ 1 minute and HAT_2_CH_2_ score ≥ 3 for neurologic events. The patients with both AHRE ≥ 1 minute and HAT_2_CH_2_ score ≥ 3 had the highest area of 0.898, 95% CI: 0.831-0.965, p < 0.001.

**Figure 3:**
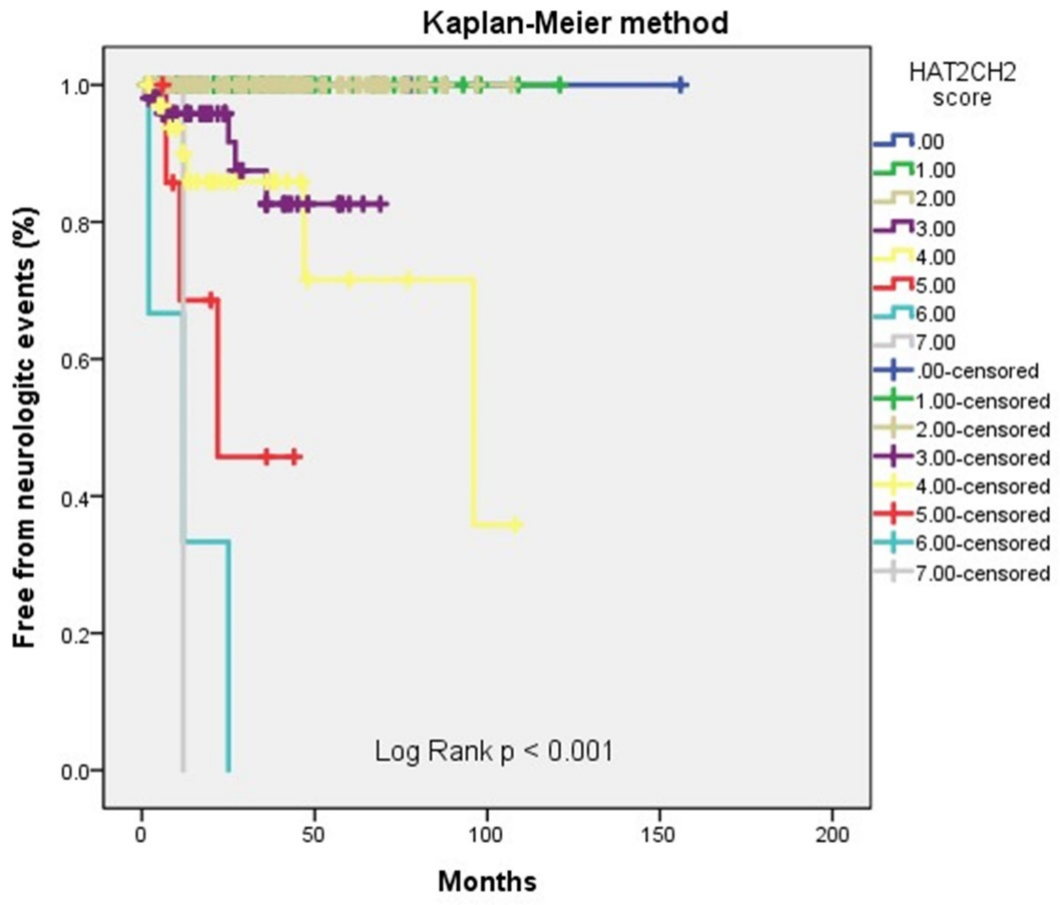
Kaplan-Meier curves depict the accumulative survival rates free from neurologic events regarding HAT_2_CH_2_ score (0-7, log-rank *p* < 0.001).

**Figure 4:**
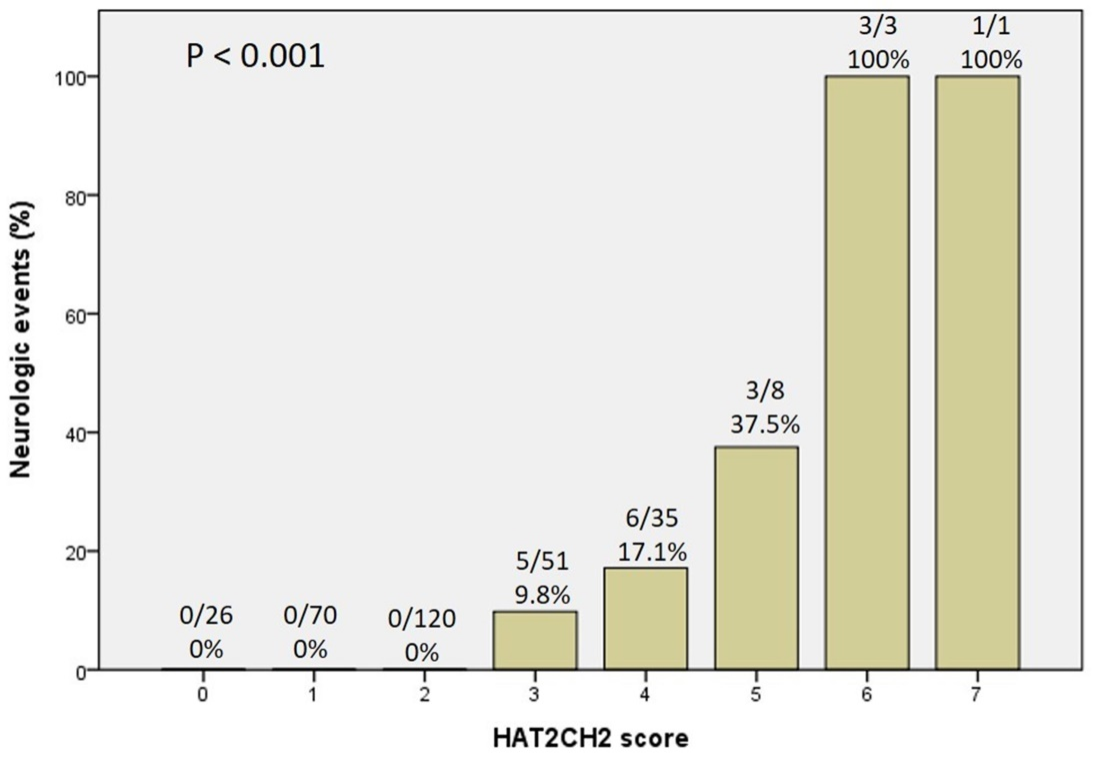
Neurologic events rate significantly increased with the HATCH score.

We further used the AHRE ≥ 1 minute and HAT_2_CH_2_ score ≥ 3 to analyze the added value of one be to the other, which showed that patients with both AHRE ≥ 1 minute and HAT_2_CH_2_ score ≥ 3 had the highest area of 0.898, 95% CI, 0.831-0.965, p < 0.001, compared to only AHRE ≥ 1 minute: area of 0.753, 95% CI, 0.665-0.840, p < 0.001; only HAT_2_CH_2_ score ≥ 3: area of 0.865, 95% CI, 0.817-0.912, p < 0.001 (Fig. 2). Kaplan-Meier curves depict the different accumulative survival rates free from NE for HAT_2_CH_2_ score groups from 0-7. The survival curve shows that patients with HAT_2_CH_2_ scores of 4-7 had higher risk for NE development compared with those with HAT_2_CH_2_ scores of 0-3 (log-rank test, *P* < 0.001) (Fig. 3). The NE occurrence percentage increased significantly with HAT_2_CH_2_ scores from 0-2 (0%), 3 (9.8%), 4 (17.1%), 5 (37.5%), to 6-7 (100%) (p < 0.001) (Fig. 4). Included patients could be further classified as very low risk with HAT_2_CH_2_ scores of 0-2, low risk with HAT_2_CH_2_ scores of 3, medium risk with HAT_2_CH_2_ scores of 4, high risk with HAT_2_CH_2_ scores of 5, and very high risk with HAT_2_CH_2_ scores of 6-7.

## Discussion

The main finding of the present study is that the HAT_2_CH_2_ score and AHRE are significantly and independently associated with NE in a Taiwanese population with CIED and no history of AF. The optimal cutoff value of the HAT_2_CH_2_ score for subsequent NE was 3 and AHRE was 1 minute. These results suggest that comprehensive assessment of the HAT_2_CH_2_ score and AHRE in patients with CIEDs is essential to support early, aggressive therapy to prevent NE.

The leading scoring systems for predicting subsequent NE after CIED implantation include the CHA_2_DS_2_-VASc score, HAT_2_CH_2_ score, C_2_HEST score and mCHEST score, often used in conjunction with the optimal cutoff for AHRE duration. Of these, the CHA_2_DS_2_-VASc score is a well-known and guideline-recommended measure (9) for NE risk prediction. However, recent studies 10,11 have reported that the CHA_2_DS_2_-VASc score has insufficient discriminative power for NE prediction. The meta-analysis of Siddiqi et al. 10 reviewed and analyzed 9 studies of patients (n = 453747) with non-valvular AF and 10 studies of patients (n = 138262) without non-valvular AF and found only a modest discriminative power using C-statistics. Similarly, Hu et al. 11 used the Taiwan Health Insurance Research Database to select and evaluate a large group of patients with venous thromboembolism (n= 56996), finding that the area under the curve of ROC of CHA_2_DS_2_-VASc score for predicting NE was 0.66, which was also modest.

In the present study, the area under the curve of ROC curve was 0.92, indicating a highly discriminative measure. To the best of our knowledge, the present study is the first to reveal that the HAT_2_CH_2_ score is superior to the CHA_2_DS_2_-VASc score, C_2_HEST score and mCHEST score in the reliable prediction of NE in patients with CIED. Further, results of the present study support our proposed risk stratification system for NE as follows: HAT_2_CH_2_ scores of 0-2 indicate very low risk, HAT_2_CH_2_ scores of 3 indicate low risk, HAT_2_CH_2_ scores of 4 indicate medium risk, HAT_2_CH_2_ scores of 5 indicate high risk, and HAT_2_CH_2_ scores of 6-7 indicate very high risk. In future study, an external validation population is needed to ensure HAT_2_CH_2_ score accuracy in predicting NE in patients with CIED.

Results of the present study also demonstrated that the HAT_2_CH_2_ score (AUC of ROC 0.65) independently predicts new onset AF in this study population (data not shown). The HAT_2_CH_2_ score includes COPD as one point, rather than diabetes or vascular disease as one point in the CHA_2_DS_2_-VASc score, which highlights the varied impact of different diseases in NE in patients with CIED based on our study results. COPD-related systemic inflammation and oxidative stress may promote platelet hyperactivity and cerebral vascular dysfunction 12, and COPD increases the risk of NE, independent of other shared risk factors of cardiovascular disease 13. Smoking has been recognized as a major causative factor for COPD 14 and also a leading cause of NE 15. Additional prospective studies are required to elucidate the possible mechanisms underlying COPD-related NE risk, and then to identify effective preventive interventions.

Different cutoff values of AHRE have been proposed previously. One Japanese study even demonstrated that AHRE lasting ≥ 30 seconds was associated with increased risk of stroke 16. That study enrolled 348 patients who received CIED from Medtronic (atrial rate > 175 beats/minutes), Abbott (atrial rate > 190 beats/minutes), and Biotronik (atrial rate > 200 beats/minutes). Atrial sensitivity was programmed to 0.5 mV with bipolar sensing. Another study 17 enrolled 355 patients who received dual chamber pacemakers from Medtronic (atrial rate > 175 beats/minutes) and Biotronik (atrial rate > 200 beats/minutes) and atrial sensitivity was programmed to 0.3 mV with Medtronic bipolar sensing and 0.2 mV with Bieotronic bipolar sensing. That study also revealed that AHRE ≥ 2 minutes increased risk of NE. In the present study, all 314 patients included had been implanted with Medtronic CIED to prevent the different default settings for AHRE detection from affecting multivariate analysis; we also did the multivariate analysis using AHRE ≥ 2 minutes but the HR was lower than AHRE ≥ 1 minute (data not shown), so we concluded that the optimal AHRE cutoff was 1 minute for subsequent NE after CIED implantation. Even though the latest guidelines 9 recommend that AHRE > 5-6 minutes and > 180 bpm detected by CIED increase the risk for NE, we still suggest that physicians should confirm the default setting of AHRE after patients have undergone CIED implantation.

### Limitations

The present study has several limitations. First, this was an observational study with a relatively small number of patients with CIED in a hospital setting, and all patients were Taiwanese. As a result, causality cannot be inferred between AHRE and NE, and the presence of confounding factors cannot be denied. Also, the results may not be generalizable to other populations or locations. Second, this study did not investigate heart rhythms at the time of NE onset, which may not give a complete picture of individual patient conditions. Third, in this retrospective analysis of patient data, we could not confirm that patients had started anticoagulants due to CIED-detected AHRE, although these patients were not excluded because no significant differences were found between anticoagulants use and presence (1, 5.6%) or absence (29, 9.8%) of NE (p = 1.000), as shown in Table 1. Prospective multicenter studies with larger samples are required to confirm results of the present study. Fourth, we did not evaluate the bleeding events after the patients receiving anti-thrombotic therapy for index NE. Every physician should measure the HASBLED score to know the bleeding risk. Fifth, we did not analyze the possible competing hazards (such as death) in this study. Sixth, we did not record the history of the anti-thrombotic drugs during follow-up periods, which may be a bias in such as a retrospective study. Finally, the major limitation was retrospective, relative small case numbers, and low rates of outcome (only 18 events). The fact is that the study could not make definite conclusions.

## Conclusions

The HAT_2_CH_2_ score and episodes of AHRE lasting ≥ 1 minute are independent risk factors for NE in patients with CIED during mid-term follow-up. When AHRE ≥ 1 minute is detected in patients with CIED, long-term monitoring is advisable to detect clinical AF as well as performing comprehensive assessment of NE risk using the HAT_2_CH_2_ score. Results of the present study suggest that early detection of AHRE ≥ 1 minute and calculation of the HAT_2_CH_2_ score in patients with CIED may be warranted to support early, aggressive therapy to prevent NE.

## Supplementary Material

Supplementary tables.

## Figures and Tables

**Table 1:** Baseline Characteristics of the Overall Study Group and with/without neurologic events

Variables	All Patients (n=314)	Neurologic events		Univariate p-value
		Yes (n=18)	No (n=296)	
Age (years)	73 (62-81)	75 (68-83)	72 (61-81)	0.153
Gender				0.212
Male	194(61.8%)	14(77.8%)	180(60.8%)	
Female	120(38.2%)	4(22.2%)	116(39.2%)	
BMI^a^ (kg/m^2^)	24.6(22.5-26.3)	23.9(22.6-25.9)	24.6(22.4-26.3)	0.616
Device type				0.318
Dual chamber PM^b^	220(70.1%)	16(88.9%)	204(68.9%)	
Dual chamber ICD^c^	66(21.0%)	1(5.6%)	65(22.0%)	
CRTP^d^	23(7.3%)	1(5.6%)	22(7.4%)	
CRTD^e^	5(1.6%)	0(0.0%)	5(1.7%)	
Primary Indication				0.178
Sinus node dysfunction	141(44.9%)	13(72.2%)	128(43.2%)	
Atrioventricular block	79(25.2%)	3(16.7%)	76(25.7%)	
Heart failure/VT^f^/VF^g^	94(29.9%)	2(11.2%)	92(9.1%)	
Atrial pacing (%)	25.0 (5.8-71.4)	18.5(1.2-87.9)	25.2(6.1-70.6)	0.922
Ventricular pacing (%)	1.9 (0.2-98.3)	14.4(0.2-75.1)	1.6(0.2-98.4)	0.522
Hypertension	253(80.6%)	17(94.4%)	236(79.7%)	0.215
Diabetes mellitus	142(45.2%)	14(77.8%)	128(43.2%)	0.006
Hyperlipidemia	241(76.8%)	18(100.0%)	223(75.3%)	0.010
Chronic obstructive pulmonary disease	14 (4.5%)	1(5.6%)	13(4.4%)	0.570
Prior stroke	19(6.1%)	6(33.3%)	13(4.4%)	<0.001
Prior myocardial infarction	57(18.2%)	5(27.8%)	52(17.6%)	0.275
Heart failure				0.462
Preserved LVEF^h^	44(14.0%)	2(11.1%)	42(14.2%)	
Reduced LVEF^h^	68(21.7%)	6(33.3%)	62(20.9%)	
None	202(64.3%)	10(55.6%)	192(64.9%)	
Chronic kidney disease	108(34.4%)	9(50.0%)	99(33.4%)	0.151
Chronic liver disease	15(4.8%)	1(5.6%)	14(4.7%)	0.596
Thyroid disease	22 (7.0%)	1(5.6%)	21(7.1%)	0.950
Hemoglobin (mg/dL)	12.0(11.013.0)	11.6(10.0-12.2)	12.0(11.0-13.0)	0.184
Platelet	206 (175-229)	201(156-244)	206(181-225)	0.738
Echo parameters				
LVEF^h^ (%)	66 (53.8-73.0)	60.0 (44.3-72.0)	66.0 (54.0-73.0)	0.242
Mitral E/e'	11.0 (8.0-13.6)	11.0 (9.7-14.3)	11.0 (8.0-13.4)	0.614
LA^i^ diameter (cm)	3.8 (3.2-4.1)	3.8 (3.5-4.4)	3.8 (3.2-4.1)	0529
RV^j^ systolic function (s', m/s)	12.0 (11.0-13.6)	12.0 (10.8-14.0)	12.0 (11.0-14.0)	0.849
Drug prescribed at baseline				
Antiplatelets	121(38.5%)	12(66.7%)	109(36.8%)	0.012
Anticoagulants	30(9.6%)	1(5.6%)	29(9.8%)	1.000
Beta blockers	122(38.9%)	6(33.3%)	116(39.2%)	0.621
Ivabradine	25(8.0%)	3(16.7%)	22(7.4%)	0.164
Amiodarone	58(18.5%)	2(11.1%)	56(18.9%)	0.543
Dronedarone	4(1.3%)	2(11.1%)	2(0.7%)	0.017
Flecainide	1(0.3%)	0(0.0%)	1(0.3%)	1.000
Propafenone	13(4.1%)	0(0.0%)	13(4.4%)	1.000
Digoxin	5(1.6%)	0(0.0%)	5(1.7%)	1.000
non-DHP CCBs^k^	12(3.8%)	0(0.0%)	12(4.1%)	1.000
RAAS^l^ inhibitors	141(45.0%)	7(38.9%)	134(45.4%)	0.589
Diuretics	47(15.0%)	4(22.2%)	43(14.5%)	0.325
Statins	121(38.5%)	6(33.3%)	115(38.9%)	0.640
Metformin	50(15.9%)	3(16.7%)	47(15.9%)	1.000
SGLT2^m^ inhibitors	13(4.1%)	0(0.0%)	13(4.4%)	1.000
Follow-up duration (months)	32 (16-52)	23.5 (11.8-44.5)	34.0 (16.0-52.0)	0.069
CHA_2_DS_2_-VASc score^n^	3 (2-4)	4 (3-5)	3 (2-4)	0.005
HAS-BLED score^o^	2 (1-3)	3 (2-3)	2 (1-3)	0.002
C_2_HEST score^p^	3 (1-3)	3 (1-4)	3 (1-3)	0.114
mC_2_HEST score^q^	3 (2-3)	3(2-4)	3(1-3)	0.099
HAT_2_CH_2_ score^r^	2 (1-3)	4 (3-5)	2 (1-3)	<0.001
AHRE^s^ Duration (minutes)	0.9(0.0-30.0)	45.0(3.5-12893.7)	0.6(0.0-14.1)	<0.001
AHRE ≥ 1 minute	147 (46.8%)	17(94.4%)	130(43.9%)	<0.001
AHRE ≥ 2 minutes	127 (40.4%)	15(83.3%)	112(37.8%)	<0.001

Data are presented as medians (interquartile interval) or n (%). Non-parametric continuous variables, as assessed using the Kolmogorov-Smirnov method, were analyzed using the Mann-Whitney U test. Statistical significance is set at p < 0.05. ^a^BMI = body mass index ^b^PM = pacemaker ^c^ICD = implantable cardioverter defibrillator ^d^CRTP = cardiac resynchronization therapy pacemaker ^e^CRTD = cardiac resynchronization therapy defibrillator ^f^VT = ventricular tachycardia ^g^VF = ventricular fibrillation ^h^LVEF = left ventricular ejection fraction ^i^LA = left atrium ^j^RV = right ventricle ^k^non-DHP CCBs = non-dihydropyridine calcium channel blockers ^l^RAAS = renin-angiotensin-aldosterone system ^m^ SGLT2 = sodium glucose co-transporters 2 ^n^ CHA_2_DS_2_-Vasc score = Range from 0 to 9. History of heart failure, hypertension, diabetes, vascular disease, age 65-74 years, and female sex each is calculated as 1 point; 75 years or older and prior stroke, TIA, or thromboembolism each is calculated as 2 points. ^o^HASBLED score = Range from 0 to 9. Point score is calculated as 1 point each for hypertension, abnormal kidney function, abnormal liver function, prior stroke, prior bleeding or bleeding predisposition, labile international normalized ratio (INR), older than 65 years, medication usage predisposing to bleeding, and alcohol use. ^p^C_2_HEST score = Range from 0 to 8. C_2_: CAD/COPD (1 point each); H: hypertension (1 point); E: elderly (age ≥ 75 years, 2 points); S: systolic HF (2 points); and T: thyroid disease (hyperthyroidism, 1 point). ^q^mC_2_HEST score = Range from 0 to 8. C_2_: CAD/COPD (1 point each); H: hypertension (1 point); E: elderly (age 65~74 years, 1 point; age ≥ 75 years, 2 points); S: systolic HF (2 points); and T: thyroid disease (hyperthyroidism, 1 point). ^r^HAT_2_CH_2_ score = Range from 0 to 7. Hypertension, 1 point; age >75 years, 1 point; stroke or transient ischemic attack, 2 points; chronic obstructive pulmonary disease, 1 point; heart failure, 2 points. ^s^AHRE = atrial high-rate episodes

**Table 2:** Types and incidences of neurologic events

Types of neurologic events	Number	Incidence rate (100 patient-years)	95% CI^a^
Transient ischemic attack	11	1.31	0.81-2.63
Ischemic stroke	7	0.84	0.51-1.67
Total events	18	2.15	1.32-4.30

^a^CI = confidence intervals

**Table 3:** Multivariable Cox regression analysis of neurologic events

Variables	Model 1			Model 2			Model 3		
	HR	95%CI	*p*	HR	95%CI	*p*	HR	95%CI	*p*
Prior stroke (yes)	8.535	2.660-27.385	<0.001	9.166	3.099-27.111	<0.001	2.780	0.869-8.891	0.085
Diabetes mellitus (yes)	2.617	0.764-8.967	0.126	2.715	0.840-8.779	0.095	1.286	0.401-4.123	0.673
Hyperlipidemia (yes)	1.918	0.000-1.177	0.971	1.137	0.000-1.175	0.957	1.761	0.000-3.200	0.960
AHRE ≥ 1 minute	**17.020**	**2.235-129.6**	**0.006**	**17.686**	**2.298-136.08**	**0.006**	5.937	0.736-47.863	0.094
CHA_2_DS_2_-VASc score	**1.168**	**0.760-1.797**	**0.479**						
HAS-BLED score				1.223	0.739-2.025	0.434			
HAT_2_CH_2_ score							**2.424**	**1.683-3.492**	**<0.001**

## Abbreviations

AF: atrial fibrillation
AHRE: atrial high-rate episodes
AMI: acute myocardial infarction
CIED: cardiac implantable electronic device
COPD: chronic obstructive pulmonary disease
eGFR: estimated glomerular filtration rate
NE: neurologic events
TIA: transient ischemic attacks
