# Supplementary material for: HAT2CH2 score performance predicting neurologic events after cardiac implantable electronic device
Source: Int J Med Sci. 2022 May 21;19(5):932–40. doi: 10.7150/ijms.72497 (PMC9149640; doi:10.7150/ijms.72497)
Supplement: Supplementary file 1 — Supplementary tables. [file ijmsv19p0932s1.pdf]

Supplement Table 1. Baseline Characteristics of the Overall Study Group and with/without new atrial fibrillation

Variables	All Patients (n=314)	New atrial fibrillation		Univariate P valve
		Yes (N=28)	No (N=286)	
Age (years)	73 (62-81)	77(66-83)	72(62-80)	0.168
**Gender**				**0.024**
**Male**	**194(61.8%)**	**23(82.1%)**	**171(59.8%)**	
Female	120(38.2%)	5(17.9%)	115(40.2%)	
BMI[a] (kg/m^2)	24.6(22.5-26.3)	24.4(23.0-26.7)	24.6(22.4-26.3)	0.963
Device type				0.014
Dual chamber PM[b]	220(70.1%)	27(96.4%)	193(67.5%)	
Dual chamber ICD[c]	66(21.0%)	0(0.0%)	66(23.1%)	
CRTP[d]	23(7.3%)	1(3.6%)	22(7.7%)	
CRTD[e]	5(1.6%)	0(0.0%)	5(1.7%)	
**Primary Indication**				**<0.001**
**Sinus node dysfunction**	**141(44.9%)**	**17(60.7%)**	**124(43.4%)**	
Atrioventricular block	79(25.2%)	10(35.7%)	69(24.1%)	
Heart failure/VT[f]/VF[g]	94(29.9%)	1(3.6%)	93(32.5%)	
Atrial pacing (%)	25.0 (5.8-71.4)	34.9(7.2-62.7)	24.0(5.3-75.8)	0.838
Ventricular pacing (%)	1.9 (0.2-	15.3(0.9-40.3)	1.3(0.2-98.4)	0.215

	98.3)			
Hypertension	253(80.6%)	26(92.9%)	227(79.4%)	0.130
Diabetes mellitus	142(45.2%)	16(57.1%)	126(44.1%)	0.233
**Hyperlipidemia**	**241(76.8%)**	**27(96.4%)**	**214(74.8%)**	**0.008**
Chronic obstructive pulmonary disease	14 (4.5%)	3(10.7%)	11(3.8%)	0.119
Prior stroke	19(6.1%)	1(3.6%)	18(6.3%)	1.000
Prior myocardial infarction	57(18.2%)	7(25.0%)	50(17.5%)	0.325
Heart failure				0.483
Preserved LVEF[h]	44(14.0%)	6(21.4%)	38(13.3%)	
Reduced LVEF[h]	68(21.7%)	6(21.4%)	62(21.7%)	
None	202(64.3%)	16(57.1%)	186(65.0%)	
Chronic kidney disease	108(34.4%)	14(50.0%)	94(32.9%)	0.069
Chronic liver disease	15(4.8%)	1(3.6%)	14(4.9%)	1.000
Thyroid disease	22 (7.0%)	1(5.6%)	21(7.1%)	0.950
Hemoglobin (mg/dL)	12.0(11.013 .0)	12.0(11.0-13.7)	12.0(11.0-13.0)	0.955
Platelet	206 (175-229)	204(174-224)	206(179-231)	0.873
Echo parameters				
LVEF[h] (%)	66 (53.8-73.0)	63.0 (53.3-68.8)	66.0 (54.0-73.0)	0.410
Mitral E/e’	11.0 (8.0-13.6)	11.9 (9.0-15.0)	11.0 (8.0-13.1)	0.224
**LA[i] diameter (cm)**	**3.8 (3.2-4.1)**	**4.1 (3.5-4.5)**	**3.7 (3.2-4.1)**	**0.016**

RV[j] systolic function (s', m/s)	12.0 (11.0-13.6)	12.0 (11.0-14.0)	12.0 (11.0-13.5)	0.304
Drug prescribed at baseline				
Antiplatelets	121(38.5%)	9(32.1%)	112(39.2%)	0.466
Anticoagulants	30(9.6%)	12(42.9%)	18(6.3%)	<0.001
Beta blockers	122(38.9%)	14(50.0%)	108(37.8%)	0.205
Ivabradine	25(8.0%)	2(7.1%)	23(8.0%)	1.000
Amiodarone	58(18.5%)	10(35.7%)	48(16.8%)	0.014
Dronedarone	4(1.3%)	2(7.1%)	2(0.7%)	0.041
Flecainide	1(0.3%)	0(0.0%)	1(0.3%)	1.000
Propafenone	13(4.1%)	3(10.7%)	10(3.5%)	0.099
Digoxin	5(1.6%)	0(0.0%)	5(1.7%)	1.000
non-DHP CCBs[k]	12(3.8%)	1(3.6%)	11(3.8%)	1.000
RAAS[l] inhibitors	141(45.0%)	12(42.9%)	129(45.3%)	0.807
Diuretics	47(15.0%)	9(32.1%)	38(13.3%)	0.008
Statins	121(38.5%)	8(28.6%)	113(39.5%)	0.256
Metformin	50(15.9%)	5(17.9%)	45(15.7%)	0.770
SGLT2[m] inhibitors	13(4.1%)	1(3.6%)	12(4.2%)	1.000
Follow-up duration (months)	32 (16-52)	23.5 (11.8-44.5)	32.0 (16.0-52.0)	0.832
CHA_2DS_2-VASc score[n]	3 (2-4)	3 (2-4)	3 (2-4)	0.360
HAS-BLED score[o]	2 (1-3)	3 (2-3)	2 (1-3)	0.013
C_2HEST score[p]	3 (1-3)	3 (3-3)	3 (1-3)	0.031
mC_2HEST score[q]	3 (2-3)	3(3-4)	3(1-3)	0.053

**HAT_2CH_2 score[r]**	**2 (1-3)**	**3 (2-4)**	**2 (1-3)**	**0.007**

Data are presented as medians (interquartile interval) or n (%). Non-parametric continuous variables, as assessed using the Kolmogorov–Smirnov method, were analyzed using the Mann–Whitney U test. Statistical significance is set at $p < 0.05$.

[a]BMI = body mass index [b]PM = pacemaker [c]ICD = implantable cardioverter defibrillator [d]CRTP = cardiac resynchronization therapy pacemaker [e]CRTD = cardiac resynchronization therapy defibrillator [f]VT = ventricular tachycardia [g]VF = ventricular fibrillation [h]LVEF = left ventricular ejection fraction [i]LA = left atrium [j]RV = right ventricle [k]non-DHP CCBs = non-dihydropyridine calcium channel blockers [l]RAAS = renin-angiotensin-aldosterone system [m]SGLT2 = sodium glucose co-transporters 2 [n] CHA_2DS_2-Vasc score = Range from 0 to 9. History of heart failure, hypertension, diabetes, vascular disease, age 65–74 years, and female sex each is calculated as 1 point; 75 years or older and prior stroke, TIA, or thromboembolism each is calculated as 2 points. [o]HASBLED score = Range from 0 to 9. Point score is calculated as 1 point each for hypertension, abnormal kidney function, abnormal liver function, prior stroke, prior bleeding or bleeding predisposition, labile international normalized ratio (INR), older than 65 years, medication usage predisposing to bleeding, and alcohol use. [p]C_2HEST score = Range from 0 to 8. C_2: CAD/COPD (1 point each); H: hypertension (1 point); E: elderly (age ≥ 75 years, 2 points); S: systolic HF (2 points); and T: thyroid disease (hyperthyroidism, 1 point). [q]mC_2HEST score = Range from 0 to 8. C_2: CAD/COPD (1 point each); H: hypertension (1 point); E: elderly (age 65~74 years, 1 point; age ≥ 75 years, 2 points); S: systolic HF (2 points); and T: thyroid disease (hyperthyroidism, 1 point). [r]HAT_2CH_2 score = Range from 0 to 7. Hypertension, 1 point; age >75 years, 1 point; stroke or transient ischemic attack, 2 points; chronic obstructive pulmonary disease, 1 point; heart failure, 2 points.

Supplement Table 2. Multivariable logistic regression analysis of new atrial fibrillation

Variables	**HR**	**95%CI**	***p***
Gender (male)	2.718	0.958-7.709	0.060

Sick sinus syndrome (yes)	1.120	0.460-2.726	0.803
Hyperlipidemia (yes)	1.185	0.134-10.510	0.879
Left atrial diameter (cm)	1.714	0.851-3.451	0.131
HAT_2CH_2 score	**1.447**	**1.054-1.987**	**0.022**
